# Muscle parameters in men and oxidative stress markers

**DOI:** 10.1186/s40101-025-00385-8

**Published:** 2025-01-17

**Authors:** Michał Pietruszewski, Judyta Nowak-Kornicka, Agnieszka Żelaźniewicz, Bogusław Pawłowski

**Affiliations:** University of Wrocław, Wrocław, Poland

**Keywords:** Oxidative stress, Testosterone, Muscle strength, Handgrip strength (HGS), Oxidative handicap, Hypothesis, Reactive oxygen species, Muscle mass

## Abstract

**Background:**

The oxidative handicap hypothesis posits that testosterone-dependent traits, such as muscle mass and strength, may be costly to develop due to testosterone’s pro-oxidative properties, leading to increased oxidative stress. This hypothesis suggests that only individuals with superior biological conditions can afford these costs. This study examines the oxidative handicap hypothesis, exploring the relationship between muscle mass or handgrip strength and oxidative stress markers in men.

**Methods:**

Handgrip strength and muscle mass were measured in 179 men, with muscle mass assessed using bioelectrical impedance analysis (BIA) and handgrip strength measured using a hydraulic dynamometer. Serum testosterone levels and antioxidant capacity were measured. 8-OH-dG, 8-epi-PGF2α, and protein carbonyls were measured to evaluate oxidative stress level. Pearson’s correlation and multivariate regression analyses were performed to examine the relationships between handgrip strength, muscle mass, and oxidative stress markers, controlling for age, serum testosterone levels, and antioxidant capacity.

**Results:**

No significant correlations were found between handgrip strength and oxidative stress markers, even when controlling for muscle mass, antioxidant capacity, testosterone levels, and age.

**Conclusions:**

The study’s findings do not support the oxidative handicap hypothesis in the context of muscle parameters in men. The results suggest that testosterone-driven traits like handgrip strength or muscle mass may not necessarily incur oxidative stress costs in healthy young men, possibly due to effective compensatory antioxidant mechanisms. Factors like lifestyle, diet, and genetic predisposition, which were not controlled in this study, could also influence the observed outcomes and should be included in future research.

## Background

In the realm of research on sexual selection, exploring how biological systems manage trade-offs between growth, reproduction, and survival provides significant insights into human evolution. Signaling theory, among other applications, provides a framework for understanding the development and maintenance of traits that act as indicators of individual quality. According to this theory, sexually selected traits, perceived as attractive, serve as honest signals of an individual’s genetic fitness, health, or reproductive potential. The explanation for why these signals can be considered honest indicators of an individual’s health provide the handicap hypothesis. It suggests that attractive traits, drawing the attention of the opposite sex and playing a key role in partner selection, are costly to develop and maintain, thereby impacting biological condition [1]. High-quality individuals can afford these costs, thereby signaling their superior biological condition, which refers to an organism’s overall fitness, including its health, reproductive potential, and ability to survive and thrive in its environment. Testosterone, a crucial male sex hormone, plays a significant role in these interactions by influencing the development and maintenance of secondary sexual traits, as well as reproductive, behavioral, and metabolic traits [2]. Under this framework, testosterone-dependent secondary sexual traits are hypothesized to develop at the cost of decreased immunity or increased oxidative stress, thus acting as signals of biological condition [3–6].

The oxidative handicap hypothesis [6] assumes that testosterone has pro-oxidant properties [7, 8], so the development and maintenance of traits that depend on that hormone will come at a cost in the form of a greater risk of oxidative stress. Oxidative stress is an imbalance between reactive oxygen species (ROS) production and the body’s antioxidant capacity [9]. ROS are a natural byproduct of cellular respiration characterized by the presence of an unpaired electron making them highly reactive and unstable. For this reason, ROS can have a toxic effect on the body by damaging cellular structures such as proteins, lipids, and nucleic acids [10]. To prevent this damage, ROS are usually neutralized by antioxidants of both internal (i.e., antioxidant enzymes like SOD) and external (i.e., vitamin C) origin [11, 12]. Testosterone may impose costs by impairing the body’s ability to protect or repair cellular machinery from oxidative damage or by enhancing ROS production. Thus, testosterone-dependent, attractive characteristics may come with costs related to oxidative stress, so only individuals with high biological condition can afford to bear these costs [1]. Previous studies investigating the oxidative handicap hypothesis have shown mixed results. Some research supports the hypothesis, demonstrating positive correlations between testosterone levels and oxidative stress markers in nonhuman animals and humans, suggesting that testosterone imposes measurable pro-oxidative costs [13, 14]. Conversely, other studies have reported no significant associations, raising the possibility that compensatory mechanisms, such as enhanced antioxidant defenses, may buffer these effects in certain contexts or populations [15, 16]. The vast majority of these studies have focused on birds or fish, leading to a knowledge gap regarding the pro-oxidative role of testosterone in men. One study addressing this area in humans conducted by Gangestad et al. [17] showed a positive correlation of oxidative stress markers with fluctuating asymmetry and a negative correlation with perceived masculine and healthy appearance. Noteworthy is also the work of Grebe et al. [18], which found a negative association between self-perceived athleticism in men and morning levels of oxidative stress markers. Moreover, no research has yet been conducted on the relationship between muscle mass and strength and oxidative stress level in the context of verifying the handicap hypothesis in men. This may be particularly interesting because traits such as muscle mass and strength may be more directly influenced by a man’s current testosterone levels [19, 20] than, for example, perceived facial masculinization, which primarily develops during puberty [21, 22]. This could make it easier to observe the potential trade-offs between the development of testosterone-dependent traits and oxidative stress levels in adult men.

Muscle mass and strength are strongly sexually dimorphic [23] and enhance mating value by signaling health and genetic fitness, influencing partner selection strategies [24–26]. Muscle strength can be reliably measured with handgrip strength (HGS) and correlates positively with testosterone levels [27] as well as with other masculinization traits such as facial masculinity [28, 29], male-typical body morphology [30], 2D:4D ratio [31, 32], and personality or behavioral traits like aggression [30, 33] or dominance [34]. Muscle mass is also strongly associated with testosterone [35], masculinity [36], and male-typical sexual behavior [37]. HGS and muscle mass change throughout life, and their trajectories are similar to that of testosterone concentrations with a peak in young adulthood, followed by a decline in midlife [38–40]. Moreover, both muscle mass and strength are traits that can change relatively rapidly, so their declines may be an adaptive response of the body to harsh environmental conditions aimed at conserving energy [41, 42]. It can also be expected that muscle strength more depended on current testosterone levels rather than fetal or adolescent levels [43].

In this study, we tested the oxidative handicap hypothesis by examining the relationship between muscle mass and strength, as measured with muscle index and handgrip strength (HGS) and oxidative stress markers. According to the oxidative handicap hypothesis, only men in high biological condition will be able to bear the costs of the pro-oxidative nature of testosterone and develop higher muscularity and handgrip strength [6, 9]. Therefore, we hypothesize that men with the highest muscle index and handgrip strength values will simultaneously exhibit greater resistance to reactive oxygen species and thus incur less intracellular damage resulting from oxidative stress indicating a high biological condition. To reflect the level of oxidative stress as accurately as possible, three markers were used to indicate oxidative damage to the three main components of cellular structures: nucleic acids (total RNA/DNA oxidative damage), lipids (i8-epi-PGF2α), and proteins (protein carbonyls). Since oxidative stress is a state of imbalance between ROS production and the body’s antioxidant potential, we also controlled for total antioxidant capacity.

## Methods

### Participants and general procedure

This study was part of a broader project on factors related to men’s health. Participants were not selected based on biological condition or any specific health criteria beyond being healthy adult men. We assumed that sufficient natural variability in biological condition exists within the studied population, characteristic of WEIRD societies, to explore the relationships between testosterone-dependent traits and oxidative stress. Participants were recruited via social networks, information on the Internet (social media, university website), local newspapers, and on the radio. The recruitment and data collection procedure was approved by the Bioethics Commission at the Wrocław Medical University (number 222/2019). Each participant signed an informed consent to participate in the study and to use the obtained data for scientific purposes.

The study protocol for each participant consisted of a fasting blood draw (collected between 7:30 a.m. and 9:00 a.m.), body measurements, and answering the survey questions. Blood samples were collected by medical staff. Blood samples to separate serum were centrifuged, later portioned, and frozen at − 80 °C until analysis. A general questionnaire contained questions about demographic data, health problems, smoking, and alcohol consumption (regular, in the past 24 h, or binge drinking within the last week).

Two-hundred nine men (*M*_age_ = 35.26, *SD*_age_ = 3.49) took part in the study. Eight men were excluded due to smoking. Eight men were excluded due to the presence of chronic diseases. Four men were excluded due to high CRP levels (> 5 mg/L) or high WBC levels (> 10 × 10^9^/L), indicating ongoing inflammation. Three men were excluded due to relatively high levels of carbonylated proteins (> 3 SD). Seven men were excluded due to missing data. After all exclusions, the final sample consisted of 179 men (*M*_age_ = 35.09, *SD*_age_ = 3.49). None of the participants in the study was involved in sports professionally.

### Handgrip strength and anthropometry

Handgrip strength was measured using a handheld dynamometer (Baseline® Hydraulic Hand Dynamometers, Elmsford, NY, USA). Participants were asked to squeeze the dynamometer as hard as they could. For each hand, the measurement was taken twice. The highest of the four measurements was used in the analyses.

Overall fat mass (kg) and skeletal muscle mass (kg) were measured with bioelectrical impedance analysis (BIA). Measurements were taken using Seca mBCA 515 (Seca GMBH & Co.®, Hamburg, Germany). Body height (cm) was measured, and muscle index was calculated according to the following formula:

$$Muscle\;index=\frac{Skeletal\;muscle\;mass\;\lbrack kg\rbrack}{{Body\;height\;\left[m\right]}^2}$$

### Testosterone measurement

The total testosterone level was measured commercially from blood samples collected during the visit by the diagnostic laboratory DIAGNOSTYKA® S.A.

### Markers of oxidative stress and antioxidant capacity measured in serum

Cumulative damage of nucleic acids caused by reactive oxygen species was measured using commercially available, high-sensitivity DNA/RNA Oxidative Damage ELISA kit (Cayman Chemical®, Ellsworth, USA, cat. no. 589320) that allows the detection of the main product of oxidative RNA/DNA breakdown: 8-OH-dG. 8-epi-PGF2α was assayed with Elabscience competitive-ELISA kit (cat. no E-EL-0041). Protein carbonyls were assayed with MyBioSource ELISA kit (cat. no. MBS3802635). Total antioxidant capacity (TAC) was assayed with a Cayman ELISA kit (cat. no. 709001). The kit allows to assess of aqueous- and lipid-soluble antioxidants; thus, the combined antioxidant capacities of all its constituents including vitamins, proteins, lipids, glutathione, uric acid, etc. were assessed. Inter-assay CV is 3%, and intra-assay CV is 3.4%. Sample preparation and all test procedures were performed in accordance with the manual supplied with each ELISA kit.

To evaluate the cumulative effect of variables indicating the level of oxidative damage to cellular structures (RNA/DNA oxidative damage, isoepiprostaglandine, protein carbonyls), a new variable was created: Cumulative Oxidation Damage Index (CODI), which was the average of the standardized (*Z*-scores) values of these variables.

### Statistical analyses

The normality of the distributions of the studied variables was assessed using the Shapiro–Wilk test. Results indicated that testosterone, CODI, and TAC distributions did not conform to a normal distribution. For later correlation and regression analysis, logarithmic transformation was applied to the testosterone. For CODI and TAC, a Box-Cox transformation was utilized. All variables followed a normal distribution after the transformations.

Pearson’s correlation analysis was performed to identify the relationship between CODI and HGS, age, muscle index, and total testosterone. Similar analyses were accomplished for TAC and the same variables. To investigate the link between CODI and HGS, a multivariate regression analysis was run and controlled for age, muscle index, total testosterone, and TAC as confounders.

The relationship between variables was tested using correlational analysis and multivariate regressions. Analysis was conducted using R (R Core Team, 2023) and RStudio (Rstudio Team, 2023).

## Results

**Fig. 1:**
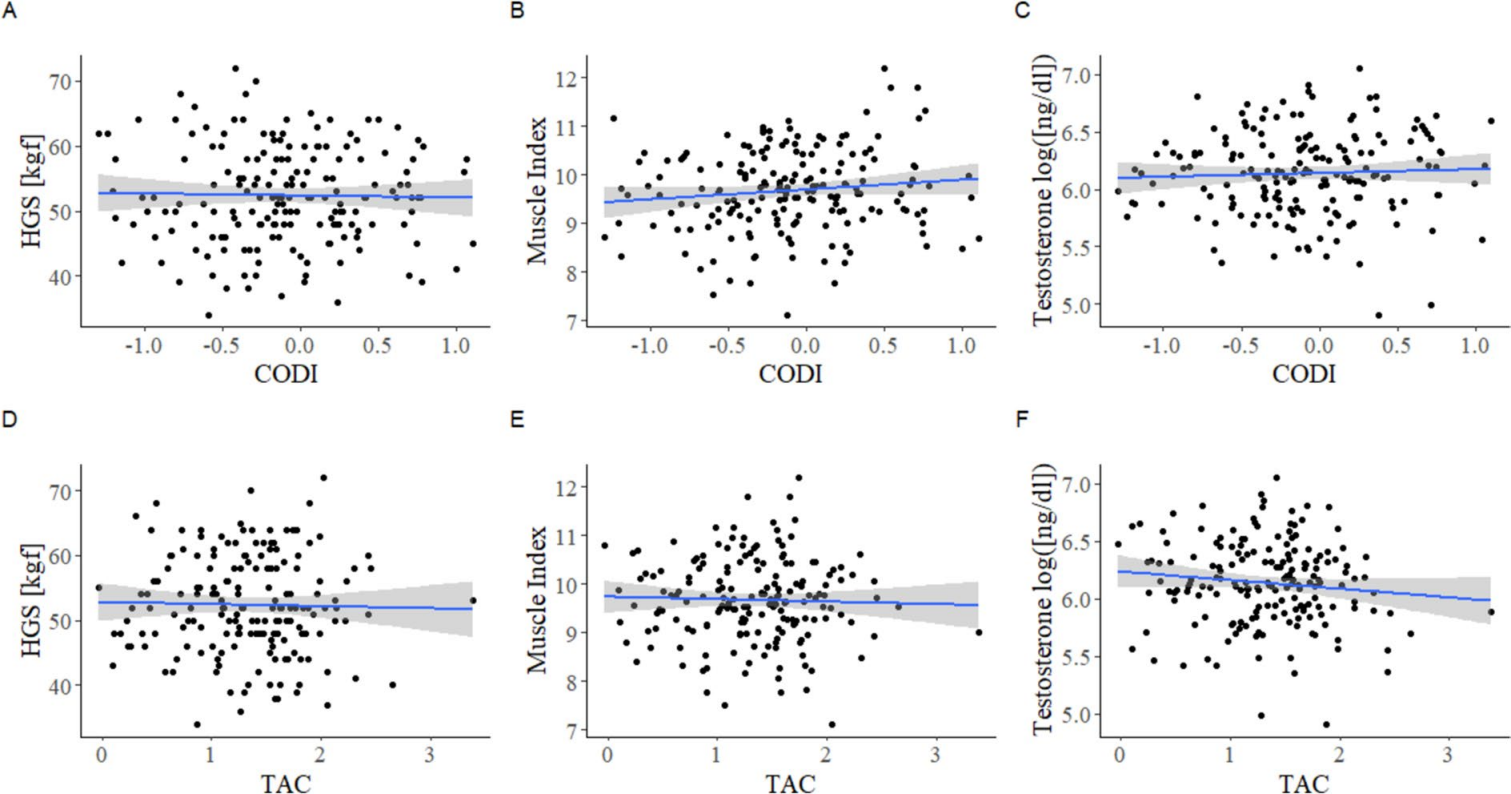
Zero-order Pearson’s correlation analyses showing that no variables associated with either CODI or TAC

Descriptive statistics for all studied variables are presented in the Table 1. Zero-order Pearson’s correlation analyses showed that none of the variables studied was associated with either CODI or TAC (Table 2, Fig. 1). Muscle index was positively associated with testosterone (ng/dl; log) (*r*_(177)_ =  − 0.22, *P* = 0.003) and with HGS (kgf) (*r*_(177)_ = 0.38, *P* < 0.001). However, there was no correlation between HGS (kgf) and testosterone levels (ng/dl; log) (*r*_(177)_ = 0.02, *P* = 0.781).

**Table 1:** Descriptive statistics (*N* = 179)

	*M*	SD	Min	Max
Age (years)	35.09	3.49	26.60	44.30
HGS (kgf)	52.40	7.58	34.00	72.00
Muscle index	9.67	0.86	7.12	12.20
Testosterone (ng/dl)	492.45	176.91	135.30	1157.00
8-epi-PGF2α (pg/ml)	207.78	68.51	97.53	414.42
RNA/DNA (ng/ml)	17.38	6.76	6.20	37.79
Protein carbonyls (ng/ml)	31.70	11.50	2.85	72.40
CODI	− 0.03	0.49	− 0.84	1.47
TAC (µm)	2.02	0.38	0.97	3.20

**Table 2:** Results of correlation analyses for the relationship between CODI, TAC, and the predictors (*N* = 179, *df* = 177)

	**CODI**^**a**^			**TAC**^**a**^		
	*r*	*p*	*CI*	*r*	*p*	*CI*
**HGS (kg)**	− 0.02	0.78	− 0.17; 0.13	− 0.02	0.75	− 0.17; 0.12
**Age (years)**	0.01	0.87	− 0.13; 0.16	− 0.05	0.51	− 0.19; 0.10
**Muscle index (kg/m**^**2**^**)**	0.12	0.11	− 0.03; 0.26	− 0.03	0.66	− 0.18; 0.11
**Testosterone log([ng/dl])**	0.05	0.55	− 0.10; 0.19	− 0.11	0.13	− 0.26; 0.03

^a^Data after Box-Cox transformation

Multivariate regression analyses for the relationship between CODI and HGS, controlled for age, testosterone, and TAC as cofounders, showed no significant relationship, and the overall model was not significant (*R*^2^ = 0.004, *F*_(4174)_ = 1.55, *P* = 0.95, Table 3). Due to the high collinearity between muscle index with testosterone (ng/dl; log) and HGS (kgf), muscle index was excluded from this model. The second model, examining the relationship between CODI and muscle index, controlling for age and TAC showed no association (*R*^2^ = 0.015, *F*_(3175)_ = 0.86, *P* = 0.46). The results of this model are presented in Table 4.

**Table 3:** Relationship between the CODI^a^ and HGS [kgf], controlling for confounders (*N* = 179)

	*B*	t(174)	*p*
Age (years)	− 0.58	0.27	0.48
HGS (kgf)	− 0.002	− 0.32	0.75
TAC (µm)^a^	0.02	0.33	0.74
Testosterone log([ng/dl])	0.07	0.11	0.51

^a^Data after Box-Cox transformation

**Table 4:** Relationship between the CODI^a^ and muscle index, controlling for age and TAC (*N* = 179)

	*B*	t(175)	*P*
Age (years)	− 0.002	− 0.16	0.88
Muscle index (kg/m^2^)	0.07	1.58	0.12
TAC (µm)^a^	0.02	0.30	0.76

^a^Data after *Box-C*ox transformation

## Discussion

In the present study, no relationships between testosterone, muscle parameters, and oxidative stress were observed in men within the studied age range. Our findings do not support the predictions of the oxidative handicap hypothesis, which suggests that testosterone exerts substantial prooxidative effects that can be sustained only by individuals of a higher biological condition. Consequently, testosterone-depended traits should reflect an individual’s ability to manage oxidative stress [9]. Our study highlights inconsistencies with previous work in this area. For instance, Grebe et al [18]. found that higher social dominance and athleticism (i.e., testosterone-dependent traits) are associated with lower oxidative stress (assessed based on 8-OHdG levels). However, Foo et al. [44] reported no significant relationship between perceived facial masculinity and oxidative stress. Most of the previous research on the oxidative handicap hypothesis has focused on nonhuman animals such as fish and birds [9, 45]. Moreover, in these animals, researchers primarily focused on such testosterone-dependent traits, as colored plumage on feathers and the skin, which are mainly produced by the deposition of carotenoids. Carotenoids have strong antioxidant properties and are thus (contrary to muscle mass and strength in man) directly related to the trade-offs between developing these traits and the body’s oxidative stress [16, 46]. Importantly, even when controlling for total antioxidant capacity (TAC) in our analysis, the results remained unchanged, which additionally challenges the hypothesis of oxidative handicap.

We hypothesized that individuals who were able to develop high muscle strength and mass as testosterone-dependent traits would also be able to bear the cost of testosterone in the form of high oxidative stress, according to the oxidative handicap hypothesis. However, our results do not confirm the hypothesized negative relationship between muscle parameters and oxidative stress markers. Nonetheless, this does not necessarily imply inconsistency with the assumptions of the oxidative handicap hypothesis, and the observed values may result from physiological trade-offs. The result of our study may suggest that either testosterone does not exert a significant pro-oxidative effect, or, at least in the men in the studied age range, the body effectively neutralizes potential oxidative stress resulting from investment in testosterone-dependent traits through compensatory antioxidant mechanisms. More muscular and stronger men (with high HGS) may have antioxidant systems efficient enough to prevent oxidative stress allowing them to develop higher muscularity. In contrast, men with a lower ability to deal with reactive oxygen species are unable to develop high muscularity and strength keeping their oxidative stress low. As a result, both men with high muscle parameters and those with low muscle parameters may have similar values of oxidative stress markers, making the relationship between muscle parameters and oxidative stress difficult to detect. In such a case, one may expect that the relationship between muscularity and TAC (rather than OS markers) should be observed. However, we also found no relationship between muscle strength or mass and TAC levels. Our observations could also align with other studies that have suggested that the relationship between testosterone and oxidative stress may be modulated by factors such as lifestyle, diet, and genetic predisposition, which were not controlled for in this study [7, 47]. In particular, regular physical activity can modulate the balance between reactive oxygen species production and antioxidant capacity, in favor of the latter [48]. This is especially important when examining traits like muscle mass and strength, as consistent physical activity not only supports muscle development and function but also boosts antioxidant defenses. These factors could buffer the effects of ROS generated through normal activities, thus maintaining oxidative stress levels within a narrow, relatively stable range regardless of variations in muscle strength or testosterone levels [49]. Therefore, future studies aiming to verify the oxidative handicap hypothesis should include more detailed control of various factors related to oxidative balance, including diet, supplementation, physical activity, and sleep patterns. However, TAC level, similarly to OS markers, may change depending on various conditions independently of muscle mass or strength [48, 50], which may also explain the lack of association between muscle parameters and TAC level observed in our study. It is also worth mentioning that there is inconsistency in research regarding the pro-oxidant properties of testosterone. Some studies confirm testosterone’s pro-oxidant properties, but some studies indicate that testosterone may have antioxidant effects under certain circumstances [51, 52].

It is also plausible that some cost of developing greater muscularity may be visible later in life. Such an effect has been shown in women, where higher perceived facial attractiveness in reproductive age was related to higher oxidative stress in the postmenopausal period [53]. Possibly, a similar effect may be detected in men, and the higher muscle mass and strength in reproductive age may be costly due to increased oxidative damage that can be detected in older age when protective mechanisms are less effective.

Studies addressing the oxidative handicap hypothesis are very diverse in their methods, assumptions, research organisms, markers used, and selected control factors. Much of the research highlights significant challenges in comparing and analyzing oxidative stress levels due to the high variability of oxidative stress markers which are influenced by various factors related to health status, environmental condition, age, chronic diseases, etc. [54–56]. Although the present study was conducted on a relatively large group of men for this kind of research, the various factors influencing oxidative stress levels and their dynamic changes over time may render the sample insufficient for statistical analyses to detect significant relationships. Moreover, our research sample was highly homogeneous, consisting solely of adult, healthy men, undifferentiated within their biological condition, mostly residing in large cities, and not engaged in professional sports. Stable environmental conditions might hinder the observation of the relationship between testosterone-dependent traits and markers of oxidative stress, due to fewer compromises between different bodily functions that could lead to more pronounced deficiencies between them. While this design aimed to capture the natural variation in these traits and their associated costs without confounding factors such as chronic illnesses or environmental stress, it also limited the variability necessary to detect the full range of potential trade-offs predicted by the oxidative handicap hypothesis. Future research should aim to include participants with diverse health statuses, ranging from individuals with optimal biological condition to those under physiological or environmental stress. Such stratification would allow for a more nuanced analysis of how testosterone-dependent traits interact with oxidative stress markers across a broader spectrum of biological conditions. In this study, we used handgrip strength measurement as an indicator of muscle functionality, a widely used measure in clinical practice. However, measuring the strength of other body parts, such as the back or leg strength, could also be beneficial for future analyses in more accurately assessing muscle functionality. Also, the measurement of body composition using bioelectrical impedance may not reflect accurate body composition, as the effectiveness of this measurement is influenced by a number of confounding factors. This limitation could partially explain the lack of significant findings in our study. Therefore, it may be worth considering in future research using several methods of body composition measurement (i.e., dual-energy X-ray absorptiometry (DEXA)), simultaneously to achieve the most reliable outcome [57].

Given the unexpected nature of the findings, further research is warranted to explore these relationships in different populations, such as professional athletes or older adults, where the interplay between testosterone, muscle strength, and oxidative stress may be more pronounced. Longitudinal studies would be particularly valuable in providing insights into how these relationships evolve, especially in response to changes in physical activity levels and overall health status. Since the study was conducted on a relatively homogeneous sample, it might be necessary to test our hypotheses in a population living in a more harsh environment, where the trade-offs resulting from the prooxidant nature of testosterone would be more visible.

## Conclusions

The findings of this study do not support the oxidative handicap hypothesis in the context of muscle strength in men, but they also do not allow us to reject it. Despite comprehensive analysis, no significant relationship was found between handgrip strength and oxidative stress markers. This suggests that compensatory mechanisms in healthy adult men may affect pro-oxidative effects associated with testosterone-dependent traits. These results emphasize the need for further research in more diverse populations, controlling factors important in shaping oxidative stress, such as lifestyle or genetic predispositions.

## Data Availability

The datasets used and/or analyzed during the current study are available from the corresponding author on reasonable request.

## Abbreviations

BIA: Bioelectrical impedance analysis
CODI: Cumulative Oxidation Damage Index
CRP: C-reactive protein
ELISA: Enzyme-linked immunosorbent assay
HGS: Handgrip strength
ROS: Reactive oxygen species
SOD: Superoxide dismutase
TAC: Total antioxidant capacity
WBC: White blood cells
